# Sustainable Access to Antimicrobials; A Missing Component to Antimicrobial Stewardship—A Tale of Two Countries

**DOI:** 10.3389/fpubh.2018.00324

**Published:** 2018-11-14

**Authors:** Lucille Malan, Quinten Labuschagne, Erich Brechtelsbauer, Debra A. Goff, Natalie Schellack

**Affiliations:** ^1^Division of Clinical Pharmacy, School of Pharmacy, Sefako Makgatho Health Sciences University, Ga-Rankuwa, South Africa; ^2^Department of Pharmacy, Emory Healthcare, Atlanta, GA, United States; ^3^Department of Pharmacy, Wexner Medical Center, The Ohio State University, Columbus, OH, United States

**Keywords:** antimicrobial resistance, antimicrobial stewardship, stockouts, pharmacist, international collaboration

## Abstract

Antimicrobial stewardship encompasses a wide range of processes and interventions designed to ensure that antimicrobials are used in the most effective manner. An important, but often neglected process to include is medicine procurement within hospitals, as the untimely administration of antimicrobials has a direct impact on patient care and antimicrobial resistance. A tender system (an open Request for Proposal, RFP) in South Africa is used in the procurement process to supply medicines and pharmaceutical supplies, whereas in the United States, each hospital is responsible for their own procurement processes. Possible key challenges facing countries to ensure a sustainable medical procurement are poor procurement practices, outdated information systems, and unavailability of human resources to support the current system. This article describes the need for adequate pharmacist–led inventory management systems, for sustainable antimicrobial delivery and the successful implementation of antimicrobial stewardship programs. Strategies to improve inventory control and medication delivery for public sector hospitals in South Africa include the development of national pharmaceutical data management standards. Pharmacist involvement in inventory management principles will ensure that antimicrobials are consistently in adequate supply for patient use, hence promoting safe and effective use while decreasing antimicrobial resistance.

## Introduction

The Global Action Plan (GAP) on antimicrobial resistance (AMR) provides a “One Health” blueprint for national action plan development and calls for enhancing the use of antimicrobial medicines in human and animal health through surveillance and research (1). The GAP provides the framework for a national action plan to combat AMR. Special emphasis is placed on strengthening of existing principles of Antimicrobial Stewardship (AMS) (2).

A missing component of AMR governance is medicine procurement and inventory control. Antimicrobial stewardship programs (ASP), which aim to optimize the use of antimicrobials, cannot succeed without involvement of medicine procurement to ensure the timely availability of antimicrobials. Essential medicine shortages are a growing problem worldwide stressing global health systems, increasing cost, and jeopardizing patients who are in direct need of essential medicines (3) especially for multidrug resistant bacteria, fungi, Human Immunodeficiency Virus (HIV), and Tuberculosis (TB) due to the ubiquitous expansion of AMR (4). The latter is also true for parts of South Africa (SA), where access to antimicrobials rather than the excessive use of antimicrobials is a challenge. The overall stock out in healthcare facilities across SA of at least one ARV or TB medicine increased by 15% from 2013 (5). Medicine shortages are a complex and global phenomenon, being reported in countries of all income levels (6). An inquiry of United States (US) health care workers showed that in up to 99% of surveyed hospitals, medicine shortages were experienced in the months preceding this study, affecting every stakeholder of the health care sector (7, 8). Antibiotics like Piperacillin-tazobactam and Benzathine penicillin (BPG) are some short-listed drugs in tight supply, in multiple countries (9), like Germany and Brazil. Antimicrobial shortages can have especially dire consequences, since doctors have to resort to sub-optimal treatments that are less effective and have distressing consequences that contributes to the rise of AMR (10, 11).

Management of infections requires the sufficient supply of antimicrobials at affordable prices. The latter can only become a reality with comprehensive procurement systems and robust supply chain management systems (4). Within sub-Saharan Africa, SA still has one of the highest disease burdens, with a high prevalence of HIV/AIDS, as well as TB, resulting in a 122nd rank out of 188 countries in terms of the Sustainable Development Goals in 2016 (12, 13). Approximately 7–8 million South Africans have HIV/AIDS (14, 15). The South Africa's National Strategic Plan For HIV, TB, and STIs 2017–2022 (16), reports that the number of MDR-TB cases doubled from 2007 to 2012. The high prevalence of patients with HIV/AIDS and TB in SA contributes to the poor outcomes of these patients when they also acquire a multidrug resistant (MDR) bacterial infection. The South African Antimicrobial Resistance Strategy Framework reports over 50% of hospital-acquired *S. aureus* isolated from the blood in 2010 were found to be MRSA (17), and up to 75% of *K. pneumoniae* isolated from the blood of hospitalized patients were extended spectrum beta-lactamase (ESBL) producing (17). These challenges have a profound effect on providing consistent supply of antimicrobials to the South African population. Antimicrobial use drives AMR. Stockouts in SA has been especially prevalent in the antiretroviral (ARV) and medicines used in TB, which might be related to the large population served in the country (5).

There is however an appreciable number of ongoing programmes that improve the quality of care within public hospitals in SA, as well as a reduction in current AMR rates (4). Reduction of AMR rates is critical since the antimicrobial utilization has globally increased with 36% between 2000 and 2010 and part of them are inappropriately used. Countries such as China, India, and SA account for 76% of this increase (18). Increasing AMR rates pose a serious public health threat, including increasing healthcare costs (18–20). Given the facts, programmes and activities aimed at improving the pharmaceutical supply chain through stabilizing inventory and obtaining essential medicines at low prices are key for effective and sustainable AMS.

This paper aims to emphasize the importance of pharmacist-involvement in the pharmaceutical supply chain to ensure and sustain adequate supply of antimicrobials as part of AMS, having a direct impact on the AMR issues described. Bridging the connection between AMS and inventory management principles will support the acquisition of medications free from adulteration and tampering, and will ensure that antimicrobials are in adequate supply for patient use, thus promoting safe and effective use. As a piece of the stewardship puzzle, implementing and strengthening ongoing programmes responsible for effective antimicrobial supply chain management practices will have a profound impact on preventing resistance and improving patient outcomes.

### Antimicrobial stewardship

AMS refers to coordinated interventions designed to improve and measure the appropriate use of antimicrobials by promoting the selection of the optimal antimicrobial regimen, dose, duration of therapy, and route of administration (21). It is a multidisciplinary, systematic approach that purposes to improve patient outcomes and limit the emergence of resistant pathogens while ensuring patient safety. Through its national strategic plan for AMR, SA has national core standards for antimicrobial stewardship (and for infection prevention control) that will be monitored by the Office of Health Standards and Compliance (2, 22). ASPs are designed to ensure that antimicrobials are used in the most effective manner (23), however an important, but often neglected process to include is medicine procurement within hospitals as the untimely administration of antimicrobials has a direct impact on patient care (24).

However, to improve the timely administration of antimicrobials, the medicines should be readily available in sufficient quantities. There is a need to ensure patients have a dependable supply of the right medicines, available at the right time, in the right quantity, and at the right place. Thus, it is imperative that the management of drug supply follows a stringent process. Managing the pharmacy inventory is a critical component of the medication use process for healthcare delivery. When pharmacies do not appropriately manage their inventories, they run the risk of not being able to provide sufficient medications to patients in need (24–26).

Maintaining an adequate supply of safe medications from reputable distributors is also important in today's dynamic healthcare climate. The World Health Organization (WHO) estimates that the supply of substandard medications from counterfeit markets is worth up to $75 million dollars per year (27). The authenticity and integrity of medications cannot be guaranteed when procured from untrustworthy sources, which can lead to ineffective therapy and diminished patient outcomes.

Shortages of key antimicrobials are on the rise according to a US study (28). Almost half of the shortages involved antibiotics used to treat high-risk pathogens, including *C. difficile*, carbapenem-resistant *Enterobacteriaceae* (CRE), methicillin-resistant *Staphylococcus aureus* (MRSA), and *Pseudomonas aeruginosa*, among others (28). In SA the procurement and distribution of medicines also remain inadequate (29) as indicated in a national audit of healthcare facilities published by the Health Systems Trust (HST). Measuring “availability of medicines and supplies” the Eastern Cape Province's clinics' compliance score was 54% in 2012 (30). Furthermore, in primary health care settings across SA an extremely high failure percentage of 77% was reported when measuring the “Tracer medicines as per applicable Essential Drugs List or formulary are available in the pharmacy/medicine” (30). The reported frequent stock-outs of numerous products on the Essential Medicine List (EML) including antimicrobials, indicates procurement and provision challenges hence failure of the availability of medicines and supplies (31–35). When first line antimicrobials are not available, patients may not do as well clinically or potentially even die because these antimicrobials are not available. ASPs can make recommendations and guidelines for safe and appropriate alternatives and provide clinician education on alternative treatment options as well.

### South african medication supply chain

In the public health sector of SA a number of challenges are experienced regarding the medicine distribution cycle and supply management (36). The national drug policy (NDP) of SA has been adopted about 21 years ago to ameliorate access to medicines and the use thereof in both public and private health sectors. The NDP has several health, economic and national development objectives which will be discussed. The National Essential Medicines list Committee (NEMLC) and the provincial and facility-based Pharmacy and Therapeutics Committees (PTCs) are responsible for the selection of the medicines that are available for procurement in the public healthcare sector in SA. Since 1988 the procurement of medicines in the public sector has been conducted under the auspices of the coordinating committee for the provisioning of medical supplies (COMED), while the National Treasury has taken the responsibility to award all contracts. However, a Ministerial task team was established in 2009 after reports of medicine availability that deteriorated across SA. The task team assessed the state of medicine procurement in the public sector and made recommendations regarding procurement reforms. One of these recommendations was to form a central procurement agency (CPA) toward centralizing the procurement of medicines for public sector hospitals (37). This entails the procurement of medicines and pharmaceutical supplies through an open tendering process (an open Request for Proposal, RFP) where contracts are entered into between the South African National Department of Health (NDoH) and suppliers of medicines, on behalf of all provinces. With the strategic use of market intelligence, improved competition and efficiencies associated with pooled volumes, lower medicine prices can be achieved (37).

Possible key challenges affecting sustainable medication procurement are (38):

- Insufficient financial supervision
- Insufficient communication and cooperation between stakeholders
- Poor procurement practices
- Outdated information systems, and
- Unavailability/shortage of human resources to support the current system

For the past several years medicine shortages in public healthcare facilities was due to stock-outs and depleted budgets (38). Causes for medicine shortages include; failure by provinces to pay suppliers, failure of suppliers to meet their tender quotas and the lack of expertise to monitor activities along the supply chain (38). Some of the above mentioned key challenges may be overcome with new innovations that have been piloted and implemented in SA. Financial supervision in the supply of medicines may be addressed by implemented policies that ensures standardized contracting systems. Furthermore, communication between stakeholders, procurement practices and information systems is addressed by the establishment of a National Surveillance Centre, an innovative early warning system implemented in selected pilot areas in SA, where dashboards displays medicine stock levels at primary health care facilities, hospitals and suppliers all around the country. With the use of mobile applications this system forecasts warnings where shortages are probable, the initial focus is on HIV and TB medicines, including vaccines (4). The recent health systems implemented by the NDoH strengthens five core medicine value chain functions namely, selection, contracting, supply chain management, contract management and medicine use. The latter health systems aims to improve medicine availability and use in SA (4).

The overall management of medicines, ensuring their availability at an affordable price, while maintaining quality throughout the medicine supply chain forms part of the responsibilities of a pharmacist. Hence, once medicines are procured and available at the health care facility, no additional factors (e.g., cost) will impede the medicines from being administered. A position statement by the South Africa Society of Clinical Pharmacists (SASOCP) also highlights the pharmacist's role to lead and drive the antimicrobial stewardship initiative, including evaluation of the appropriateness of prescribed antimicrobials for given indications, as well as measurement and tracking of stewardship-initiated interventions and compliance to institution-specific guidelines in their scope of practice (39). It is imperative to make use of existing human resources in SA, including pharmacists, registered nurses (RNs) as well as other health care team members to collaborate anti-infective management due to the scarcity of infectious disease (ID) specialist and epidemiologist, especially in the public sector (39).

**Figure 1:**
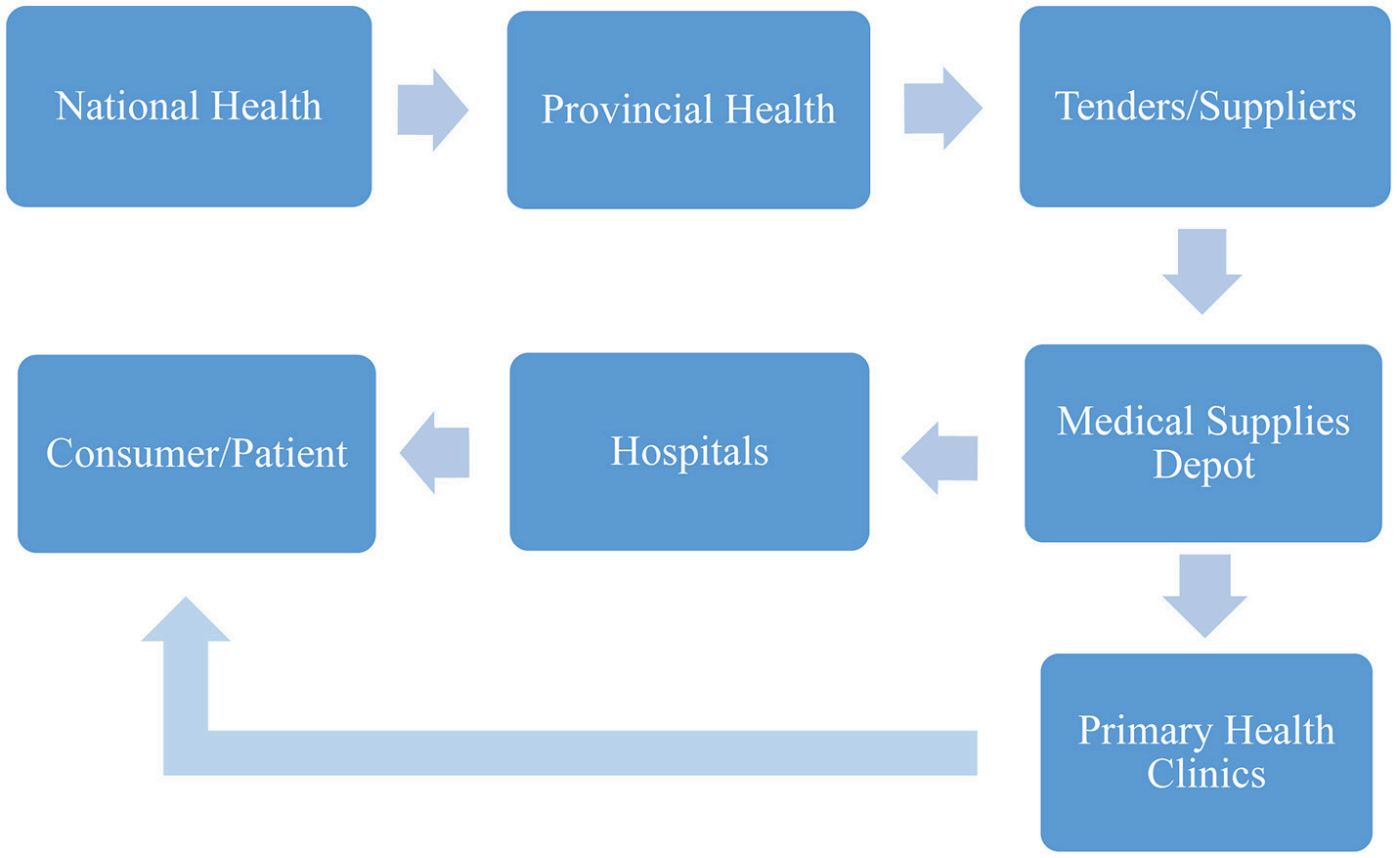
End-to-end pharmaceutical supply chain steps in SA (34).

Furthermore, the hospital pharmacists have a role in ensuring overall medicine safety, as it is their responsibility to oversee the complete medicine distribution cycle. This cycle begins with the prescription according to diagnosis, selection of medicine, dispensing, preparation and all the way through to the administration and disposal of the medicines (40). Figure 1 shows the end-to-end supply chain of pharmaceuticals in SA (41).

### United states medication supply chain

In the US, the medicine supply chain for medication procurement is relatively safe due to legislation passed by the US Congress in 2013 titled the Drug Supply Chain Security Act (DSCSA). Over a time period of 10 years, the DCSCA will require the pharmaceutical supply chain to implement medication tracking and tracing; serialization; verification and detection of suspicious products; and strict guidelines for wholesaler licensing and reporting. Through ensuring that medications are free from adulteration and tampering, the DSCSA protects patients and ensures for quality and safety within the medication procurement process (42).

Hospitals and health-systems in the US are responsible for their own medication formularies and medication procurement. In the US, there are interdisciplinary committees consisting of pharmacists, physicians, nurses, and other professionals who make up the formal Pharmacy and Therapeutics (P&T) committee. P&T committees are responsible for analyzing medication use within hospitals (43). A major function of the P&T committee is formulary management. This committee reviews all antimicrobial requests for formulary addition, compares it to the supporting evidence-based literature, and makes a determination if it will be added to the hospitals formulary of approved medications.

**Figure 2:**
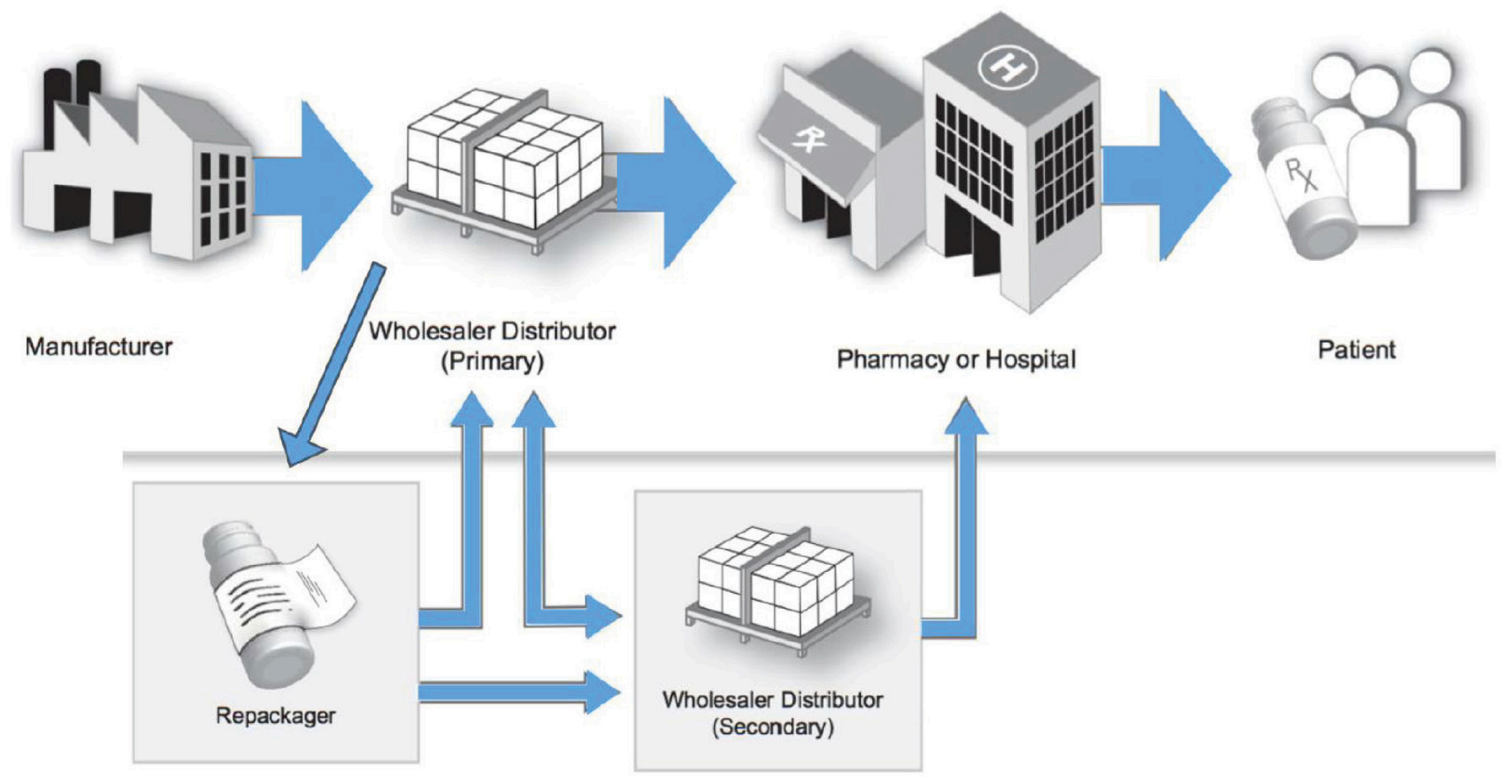
Multiple steps in the United States' pharmaceutical supply chain (37).

Once a medication is approved for use within the hospital, the medication can then be procured by the hospital's department of pharmacy. The logistics for medication purchasing and procurement depends on the wholesaler with whom the hospital pharmacy holds a contract with. A medication wholesaler serves as the intermediary between the pharmaceutical manufactures and the terminal dispenser (i.e., hospitals, pharmacies). Wholesalers purchase medications from the manufacturer, and then subsequently sell the medications to terminal dispensers (Figure 2) (44). This is very similar to the process SA follows. Since manufactures typically do not sell directly to the pharmacy, with some exceptions, this process allows manufactures to manage fewer distribution streams, resulting in larger volume sales to fewer customers. Through the DSCSA, there is a high level of visibility and accountability through these transactions to ensure the integrity and safety of the medication. Figure 2 portrays the multiple steps in the US pharmaceutical supply chain (44).

Each fiscal year, hospitals are tasked with developing their own budget for the annual cost of drugs. Projecting the following year's drug spend is a highly detailed process of predicting patient volumes, historical drug utilization, breakthrough therapy approvals, and annual inflation rates. The American Society of Health-System Pharmacists (AHSP) publishes an annual Pharmacy Forecast which assists pharmacy managers in projecting future drug budgets.

### A way forward for inventory management in stewardship

The following are factors impeding medicine supply management and although they might seem outdated it describes common obstacles faced within SA's public health system today (45):

- The lack of infrastructure for storage and distribution of drugs
- The lack of dedicated transport to ensure constant drug supply
- Losses from expiration, theft, fraud, and inappropriate storage
- Inaccurate forecasting of drug requirements due to non–adherence to drug par levels (also known as re-order levels).

There are several possible reasons contributing to the identified challenges, one of them being the absence of pharmacists and pharmacist assistants (referred to pharmacy technicians in the US) throughout the procurement, supply, storage, preparation, and distribution processes of medicine within the public sector.

Pharmacists being the custodians of medicines should play an active role during all stages of the medication use process (46). According to the Good Pharmacy Practice Manual (GPP) by the South African Pharmacy Council (SAPC), the following could serve to improve inventory management (24):

- Ward stock levels should be calculated using a consumption-based method and agreed upon by nursing staff, using the current “*pull replenishment mechanism*” as a baseline moving forward (4)
- Stock rotation should be practiced and the pharmacist or pharmacist assistant should, on a weekly schedule, proactively monitor stock in all medicine storage areas of the hospital to assure appropriate stock inventory and beyond-use dating.
- Adequate information should be gathered to predict and prepare for incoming drug shortages and stock-outs, so that alternative therapies can be made available, minimizing impact to patient care.
- The pharmacy should process all medicine orders, in accordance with the agreed stock control system and should regulate the return of unused or unwanted stock.
- The pharmacy, as well as the wards should keep records of the quantity of medicines supplied for a minimum period of 5 years

These responsibilities are also in accordance with the Medicines and Related Substances Act, Act 101 of 1965, as amended (47). The Act endorses that the distribution of medicines within a hospital should take place under the direction and control of a pharmacist. Furthermore, for the South African context it is vital to utilize existing resources, such as pharmacists, not only to drive forward ASPs, which is already happening (48), but also to highlight their vital role in and as a way to map inventory management in AMS.

### Recommendations and conclusions

Sub-Saharan Africa still has the highest burden of infectious disease in the world (12, 13, 49, 50). Furthermore, there are increasing concerns with the global growth of AMR similarly in SA, including multidrug resistant bacteria, fungi, HIV, and TB (51).

There is growing evidence that access to high-quality healthcare substantially improves health outcomes for patients including those with infectious diseases (52). Access and quality is vital, and adequate inventory management regulated by pharmacists will lead to continued dependable medicine supply necessary for the successful implementation of ASPs. The GAP on AMR also emphasizes the critical need to enhance the use of antimicrobials, alongside strengthening of the knowledge and evidence base through continued surveillance and research.

The shortage of pharmacy personnel in SA's health sector is well-known, contributing to poor pharmaceutical service delivery, which is aggravated by the inappropriate utilization of scarce expertise and failure to match high expenditure functions with the requisite skills mix. However, curriculums for the development of mid-level pharmacy support personnel, the pharmacy technician, is in the process of being approved by the SAPC and South African NDoH. These pharmacy technicians will be able to work independently but under the remote supervision of the pharmacist, freeing up pharmacists time and enabling them to be directly involved in ASP, managing drug acquisition and the full medication use process. Furthermore, it will also be more cost-effective to train pharmacy technicians to focus on medication dispensing, while utilizing pharmacists for their clinical medication expertise. A Cost-Benefit Analysis is recommended to justify the viability and cost-savings.

Strategies to improve inventory control and medication delivery include the development of national pharmaceutical data management standards. This will not only improve visibility of transactions, but will also facilitate the ease in which information and materials are exchanged along the entire supply chain. The ease of information sharing and exchange across the supply chain is a pre-requisite for transparent visibility. This not only calls for implementation of electronic medical records, but also improved integration of software, where mobile applications or electronic systems should more frequently be used to collect information and to alert of possible medicine shortages resulting in improved management of antimicrobials. Having pharmacist presence in the wards with accountability for managing the antimicrobial stock will improve access to reliable information in a timely manner in additional to managing an efficient supply of medications. When antimicrobial shortages occur, pharmacists with dedicated time to ASP's can help develop guidelines for alternative antimicrobial treatment.

These are some fundamental ingredients to ensuring that essential antimicrobials are made available to those who require them, on time, at an affordable price and in constant and sustainable supply.

## Author contributions

All authors listed have made a substantial, direct and intellectual contribution to the work, and approved it for publication.

### Conflict of interest statement

The authors declare that the research was conducted in the absence of any commercial or financial relationships that could be construed as a potential conflict of interest.
